# Diagnostic accuracy of the International HIV Dementia Scale and HIV Dementia Scale: A meta-analysis

**DOI:** 10.3892/etm.2012.665

**Published:** 2012-08-14

**Authors:** XUEYING HU, YANG ZHOU, JIANXIONG LONG, QIMING FENG, RENSHENG WANG, LI SU, TINGTING ZHAO, BO WEI

**Affiliations:** 1 School of Public Health;; 2 Department of Radiotherapy, The First Affiliated Hospital, Guangxi Medical University, Nanning, Guangxi Zhuang Autonomous Region 530021, P.R. China

**Keywords:** International HIV Dementia Scale, HIV Dementia Scale, meta-analysis

## Abstract

This aim of this study was to assess the diagnostic accuracy of the International HIV Dementia Scale (IHDS) or HIV Dementia Scale (HDS) for the diagnosis of HIV-associated neurocognitive disorders (HAND). A comprehensive and systematic search was carried out in PubMed and EMBASE databases. Sensitivity, specificity, Q^*^-values, summary receiver operating characteristic curves and other measures of accuracy of IHDS or HDS in the diagnosis of HAND were summarized. Summary receiver operator characteristic (SROC) curve analysis for HAND data demonstrates a pooled sensitivity of 0.90 [95% confidence interval (CI), 0.88–0.91] and overall specificity of 0.96 (95% CI, 0.95–0.97) for IHDS, the Q^*^-value for IHDS was 0.9195 and the diagnostic odds ratio (DOR) was 162.28 (95% CI, 91.82–286.81). HDS had an overall sensitivity of 0.39 (95% CI, 0.34–0.43) and specificity of 0.90 (95% CI, 0.89–0.91), the Q^*^-value for HDS was 0.6321 and DOR was 5.81 (95% CI, 3.64–9.82). There was significant heterogeneity for studies that reported IHDS and HDS. This meta-analysis has shown that IHDS and HDS may offer high diagnostic performance accuracy for the detection of HAND in primary health care and resource-limited settings. IHDS and HDS may require reformed neuropsychological characterization of impairments in accordance with regional culture and language in future international studies.

## Introduction

HIV/AIDS is a major global public health crisis, occurring in approximately 40 million adults and children (1), of whom approximately 26.6 million individuals are in Sub-Saharan Africa (1). The prevalence of neurological complications, including HIV-associated neurocognitive disorders (HAND), reportedly remain high in approximately 50% of HIV-seropositive (HIV+) individuals, irrespective of the use of combination antiretroviral therapy (cART) (2–5). The advent of cART has substantially altered the nature of these disorders, although they are now known to persist despite its widespread use (6). HAND results in several serious adverse outcomes, including increased mortality, decreased adherence to cART and decreased employment (7). In 2007, a revised classification was proposed for HIV-related CNS impairment that HAND includes asymptomatic neurocognitive impairment (ANI), minor neurocognitive disorder (MND) and HIV-associated dementia (HAD) (8). The diagnosis of HAND is dependent upon clinical history and neuropsychological testing in accordance with criteria developed by the American Academy of Neurology (AAN) (9). Neuropsychological testing is a critical component of the diagnosis, however, it is time-consuming, language- and education-dependent and often not available in the majority of developing countries.

A number of brief screening tools have been developed for clinical use in primary health care and resource-limited settings. These screening tools include the Mini-Mental State Exam (MMSE) (10,11), the International HIV Dementia Scale (IHDS) (12–15) and the HIV Dementia Scale (HDS) (14,16–21). However, the MMSE was designed to screen for cortical dementia such as Alzheimer’s disease. IHDS and HDS were designed to facilitate the recognition of patients at risk from HAND, but the performance accuracy varies. In addition, many of these published studies have been based on small numbers of patients. Furthermore, observational epidemiological studies are prone to bias. Therefore, we conducted this meta-analysis to pool the diagnostic performance of IHDS and HDS in detecting HAND.

## Materials and methods

### Study source

A comprehensive and systematic search throughout January, 2012, was conducted in PubMed and EMBASE databases with the key words ‘International HIV Dementia Scale’, ‘HIV Dementia Scale’ and ‘HIV associated neurocognitive disorder’. No restrictions were imposed. Additionally, we reviewed the reference lists of retrieved manuscripts and all relevant review studies. Studies were considered eligible if they met the following criteria: i) neurological and neuropsychological assessments or the modified American Academy of Neurology HIV-dementia criteria as the reference standard, ii) IHDS or HDS used as the diagnosis method and iii) HAND as a research topic. The exclusion criteria were: i) overlapping articles, ii) reviews, meta-analyses, comments and letters, iii) other methods for detecting HIV-associated neurocognitive disorder, including the MMSE or Executive Interview (EXIT), iv) the research topic was not HIV-associated neurocognitive disorder but stroke and v) only HIV-seronegative volunteers detected from the abstracts and titles of identified articles.

### Data extraction and study design assessment

Two reviewers (Hu X. and Zhou Y.) independently extracted data complying with the above-mentioned criteria and discordance was subsequently reviewed and resolved through discussion and a consensus meeting with a third reviewer (Su L.). We extracted sensitivity and specificity data that corresponded to a cut-off score of ≤10 when available. The extracted data included first author, date of publication and patient characteristics. The following characteristics of included studies were recorded: i) data for sensitivity and specificity of IHDS or HDS and ii) the quality of each included study was appraised using the QUADAS (maximum score, 14; a tool for the quality assessment of studies of diagnostic accuracy) checklist (22).

### Statistical analysis

HAND included ANI, MND and HAD. Sensitivity and specificity values, summary receiver operator characteristic (SROC) curves, diagnostic odds ratio (DOR) and the Q^*^ index were chosen to assess the diagnostic accuracy of IHDS or HDS in detecting HAND. The SROC curve was integrated by the independent specificity and sensitivity. The Q^*^ index, the best statistical method to reflect the diagnostic value, was subsequently obtained from the SROC curve of all included studies (Q^*^ index was defined as the point where sensitivity and specificity are equal, being the point closest to the ideal top-left corner of the SROC space) (23). The area under curve (AUC) measured test precision from the SROC curve. A perfect test had an AUC value of 1.0, yielding a 100% correct diagnosis irrespective of patient characteristics. The majority of clinical diagnostic tests had a value of 0.5–1.0. AUC values close to 1.0 indicated diagnostic accuracy.

The sensitivity and specificity data of the included studies were extracted or calculated by using 2×2 contingency tables of correct patient and HAND diagnosis. Statistical analyses were conducted with Stata version 9.2 (Stata Corporation, College Station, TX, USA) and Meta-DiSc for Windows (version 1.4). For each contrast, statistical heterogeneity was evaluated using a Chi-square-based Q-statistic, with P-values of <0.1 indicating the presence of heterogeneity among studies. Heterogeneity was quantified by the I^2^ statistic, when I^2^>50%, the heterogeneity was considered statistically significant. Given the heterogeneity with respect to both the random variation within individual studies and the variation among different studies, the random effects model was applied to compute the pooled sensitivity, specificity and DOR. The random effects models incorporated an estimate of the between-study variance and provided wider 95% CI margins.

## Results

### Study design

The search strategy yielded 796 studies from the literature databases (366 in PubMed, 430 in EMBASE). We eliminated 781 studies based on the titles and abstracts. Fifteen studies were potentially eligible and full texts were retrieved for further review (12–21,24–28). Five studies were excluded after reviewing the full text, four had no data for the detection of HAND (24–27) and one study had MMSE as the reference standard (28). Ultimately, 10 studies were included in this meta-analysis. Additionally, one study (12) detected HAND in independent populations and therefore we treated it as two separate studies. Subsequently, five studies with IHDS including 296 patients and seven studies with HDS with a total of 648 patients were available for this analysis.

### Characteristics and quality of the studies

Of all included studies, five studies with IHDS were published between 2005 and 2011, and seven with HDS were published between 1995 and 2011. The studies were appraised according to QUADAS. Patient characteristics and the individual scores are presented in Table I.

### Diagnostic accuracy

The pooled sensitivity and specificity for IHDS in detecting HAND were 0.64 (95% CI, 0.56–0.71) and 0.59 (95% CI, 0.50–0.67), respectively, and I^2^ values of sensitivity and specificity were 85.5 and 92.2%, respectively. The pooled DOR was 4.59 (95% CI, 2.53–8.33), with an overall AUC value of 0.7333 and the Q^*^ index was 0.6797.

The pooled sensitivity and specificity for HDS in detecting HAND were 0.61 (95% CI, 0.54–0.68) and 0.79 (95% CI, 0.75–0.83), respectively. I^2^ values of sensitivity and specificity were 85.0 and 90.9%, respectively. The pooled DOR was 8.73 (95% CI, 3.78-20.14), with an overall AUC value of 0.7853 and the Q^*^ index was 0.7231.

## Discussion

HIV-associated neurocognitive disorder is an severe complication in patients with HIV/AIDS for several reasons: i) it is associated with an increased risk of mortality (29,30); ii) the presence of HAND influences antiretroviral medication adherence, which is essential for the suppression of virological replication (31) and iii) it is potentially treatable with highly active antiretroviral medication (32,33). The diagnosis of HAND may then be an indication for the initiation of antiretroviral therapy, from which the patients themselves, their families and their communities potentially gain a great deal of benefit. Therefore, a sensitive and rapid screening test for HIV-associated neurocognitive disorder is essential. The accuracy of screening tools, including the Mental Alteration Test, the Executive Interview and the HIV Dementia Assessment, show great variability and are inconclusive. However, the IHDS and HDS are the most promising modalities for the evaluation of HAND in patients with HIV infection in developing countries.

In this meta-analysis, summary estimates and SROC were obtained for the diagnostic accuracy of IHDS and HDS in the detection of HIV-seropositive patients with HAND. The pooled sensitivity of IHDS (0.64) and HDS (0.61) were apparently equal. However, HDS (specificity, 0.79; DOR, 8.73) proved to be much better than IHDS (specificity, 0.59; DOR, 4.59) for the detection of HAND.

Several reasons may explain why no high sensitivity or specificity for IHDS or HDS use is observed in the present analysis. First, since most HIV/AIDS epidemic areas are resource-limited, with low levels of education or regional culture and language, the operators and patients with HIV infections potentially misinterpreted the HAND-screening tools, leading to an underestimation of the power of HAND-screening instruments. Therefore, an appropriate screening tool for HAND in future international studies requires reformed neuropsychological characterization of impairments in accordance with regional culture and language. Second, we extracted the majority of the sensitivity and specificity data that corresponded to a cut-off score of ≤10. However, Joska *et al* (15) suggested that IHDS may be more beneficial in retaining higher sensitivity when a cut-off value of ≤11 as compared to ≤10 was used, in order not to miss cases. It is conceivable that the sensitivity and specificity of IHDS in our meta-analysis was underestimated. Therefore, the IHDS is potentially a useful screening tool, although investigators should consider using the higher cut-off score of 11 in future studies. Third, of note is that IHDS was not useful for detecting mild neurocognitive disorder associated with HIV infection, as there was no difference between HIV-seropositive individuals with normal neuropsychological testing and HIV-seropositive individuals with mild impairment on neuropsychological testing. Therefore, it is reasonable that the result of this meta-analysis is not in agreement with the high diagnostic accuracy reported in certain observational epidemiological studies. It is a significant concern that individuals in resource-limited areas may have had undetected etiologies of potential cognitive impairment, including tuberculosis, malaria, syphilis, alcohol use or depression. The lack of neuroimaging to rule out a CNS opportunistic infection, malignancy or cerebrovascular disease and the lack of a cerebrospinal fluid examination to rule out a CNS opportunistic meningoencephalitis may limit the diagnosis of HAND. It is possible that the result of this meta-analysis has potential confounders.

Publication bias is a potential limitation of systematic review. Although various methods have been proposed to detect publication bias, different methods yield different estimates with respect to the direction and magnitude of the publication bias (34). In addition, studies with regard to publication bias focus on randomized trials. Publication bias potentially occurs when small studies with promising results are often published more readily than small studies with negative results, while larger studies with promising results are published more easily than larger studies with negative results. In the present meta-analysis, an assessment of whether the size of included studies was associated with results for diagnostic accuracy was used to examine publication bias and sample size was not associated with diagnostic performance accuracy. Since the limited number of data points for certain data sets was likely to have decreased the power to detect publication bias, a funnel plot analysis was not conducted.

Cost-effectiveness was not assessed in this meta-analysis, thus it requires future study.

To the best of our knowledge, this review of the evidence is the first with regard to the diagnostic accuracy of IHDS and HDS in the detection of HAND. However, several potential limitations of this meta-analysis merit caution. First, despite systematic research to identify relevant studies, it is possible that a number of studies related to our question were missed. Second, our ability to make strong recommendations for clinicians is hindered by the limited number and quality of studies, as well as by their heterogeneity in patient demographics (including country of origin), screening instrument used, patient selection methods, and educational and literacy level of participants. Third, since the included population was not homogeneous, evidence that a HAND-screening instrument is accurate in one subpopulation may not be generalized to other subpopulations. Finally, literature-based rather than individual-based meta-analyses present a potential source of bias.

In summary, this meta-analysis has shown that IHDS and HDS potentially offers high diagnostic performance accuracy for the detection of HAND in primary health care and resource-limited settings. Our report indicates the need for additional studies that address the diagnostic accuracy of IHDS and HDS for use in non-English-speaking populations seen in primary care settings, with reformed neuropsychological characterization of impairments in accordance with regional culture and language.

## Figures and Tables

**Table I:** Characteristics and quality of all included studies.

Study	Year	Country	Patients	Median age (years)	Cut-off point (≤)	TP	FP	FN	TN	Quality score (QUADAS)
IHDS										
Sacktor *et al* (12)	2005	USA	66	45	10	20	5	18	23	11
Sacktor *et al* (12)	2005	Uganda	81	37	10	20	5	25	31	11
Singh *et al* (13)	2008	South Africa	20	34	10	14	2	2	2	9
Skinner *et al* (14)	2009	Canada	33	52.7	10	10	3	7	13	8
Joska *et al* (15)	2011	South Africa	96	29.75	10	35	43	4	14	11
HDS										
Power *et al* (16)	1995	USA	101	37	10	30	7	6	58	8
Berghuis *et al* (17)	1999	USA	103	38	10	11	1	26	65	8
Smith *et al* (18)	2003	USA	90	40.9	10	17	27	7	39	8
Carey *et al* (19)	2004	USA	190	41	10	5	50	3	132	9
Wojna *et al* (20)	2007	Puerto Rico	60	36.4	13	36	5	10	9	8
Lyon *et al* (21)	2009	USA	71	16.8	10	5	1	14	51	11
Skinner *et al* (14)	2009	Canada	33	52.7	10	6	7	4	16	8

TP, true positive; FP, false positive; FN, false negative; TN, true negative; IHDS, International HIV Dementia Scale; HDS, HIV Dementia Scale.
